# Ameliorating potential and fertility enhancing activities of nutritional dietary supplementation of D-Ribose –l-Cysteine in cisplatin induced oligoasthenoteratozoospermia and seminiferous epithelium degeneration in adult male Sprague-Dawley rats

**DOI:** 10.1016/j.metop.2021.100128

**Published:** 2021-09-28

**Authors:** Sunday Aderemi Adelakun, Babatunde Ogunlade, Kingley Afoke Iteire, Oluwafemi Abidemi Adedotun

**Affiliations:** a Department Human Anatomy, Federal University of Technology, Akure, Nigeria; b Department of Anatomy, University of Medical Science, Ondo City, Nigeria

**Keywords:** D-ribose-l-cysteine, Cisplatin, Hormones, Testicular enzymes, Cholesterol

## Abstract

**Background:**

Cisplatin (CN) is considered as a cytotoxic agent and DNA synthesis inhibitor. D-Ribose-l-Cysteine (DRLC) is an active ingredient of riboceine, help cells to produce glutathione on body demand.

**Aim:**

Current study focused on ameliorating potential and fertility enhancing activities of D-Ribose–l-Cysteine in cisplatin induced oligoasthenoteratozoospermia and seminiferous epithelium degeneration.

**Materials and method:**

Sixty (60) male rats randomized into six groups of ten (n = 10) rats each. Group A (control) received 2.0 ml distilled water, group B received single dose of 8 mg/kg bwt CN, group C received 30 mg/kg bwt DRLC, group D received single dose of 8 mg/kg CN follow by 30 mg/kg bwt DRLC, group E received single dose of 8 mg/kg CN and vitamin C, group F received single dose of 8 mg/kg cisplatin follow by DRLC + Vit.C for 56 days. Parameters tested include: Sperm parameters, testosterone (TT), luteinizing hormone (LH), Follicle stimulating Hormone, (FSH), Prolactin, and testicular 17β-HSD activity, Blood hydroperoxide (BHP), Malondialdehyde (MDA), Superoxide Dismutase (SOD), Glutathione (GSH) and Catalase (CAT), testicular histology and fertility test.

**Results:**

Cisplatin significantly reduced testicular volume, body weight, sperm quality, fertility indices, TT, FSH, LH, 17β-HSD, SOD, GSH, CAT, diameter and cross-sectional area of seminiferous tubules, spermatogenesis score. And elevate prolactin, testicular injury score, BHP and MDA compared with control group. Cisplatin only treated rats showed degenerated seminiferous epithelium with empty lumen. Intervention of D-Ribose-Cysteine ameliorated toxic impacts of CN on testis and improved the male fertility.

**Conclusion:**

D-Ribose-l-Cysteine therefore, preserves testicular integrity and functions thereby, preventing the deleterious impact of CN.

## Graphical abstract

Schematic illustration of experimental design and administration.

**Figure:**
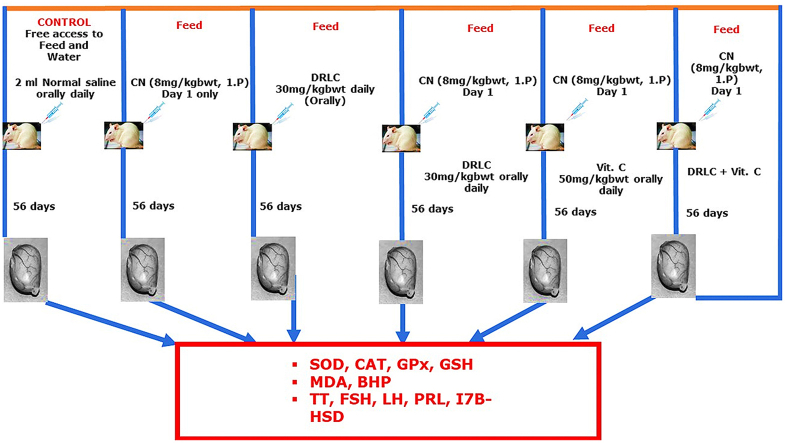

## 1 Introduction

Infertility is one of the major problems following chemotherapy in cancer treatment [1]. Chemotherapy involves uses of chemicals (anticancer drugs) to target cancerous or rapidly dividing cells [2]. Infertility has long been focusing on women, but men also, have a significant role in the ability of a couple to achieve pregnancy [3]. Cisplatin, a Platinum based antineoplastic medication with the IUPAC name (SP-4-2) diamminedichloroplatinum (II) is use in treatment of a wide variety of cancers and it has been reported to cause testicular toxicity in patients undergoing chemotherapy [4]. In young male patients, chemotherapy regimens affect fertility by influencing the testicular function. Although in cancers controlled by chemotherapy, the ability to have a normal child as a factor of life quality is a major issue. Testicular dysfunction is the most common long-term side effects of chemotherapy in men [5]. In the case of male infertility, the most common cause is sperm disorder whereby, sperm cells are produced in the testis-a process called spermatogenesis [6]. The seminiferous epithelium is made up of germ cells forming layers interspersed by somatic cells called sertoli cells [6]. Although, the sexual function may be normal, there is a reduced count of mainly dysfunctional/dysmorphical spermatozoa [6,7]. The term oligoasthenoteratozoospermia is used when three variables are present: oligozoospermia (reduced sperm count), teratozoospermia (abnormal sperm morphology) and asthenozoospermia (poor sperm motility) [3]. It is possible to broadly group the causes of OAT is link to life style factors, genetic or hereditary factors, testicular factors and testicular/ejaculatory dysfunction [8], chronic alcohol consumption, smoking, hormonal imbalance, medications, varicocoele, infections, biological or other idiopathic factors can affect the quality of semen [9]. Spermatogenesis is affected by cytotoxic drugs use in radiotherapy and cancer treatment [5,10].

Cisplatin is an antineoplastic agent which is widely use in treating various genitourinary tumors [11,12]. It is a DNA alkylating agent that exerts its anti-cancer action by numerous mechanisms, including DNA damage, apoptosis induction, and reactive oxygen species (ROS) [12]. Cisplatin is use for treatment of various malignancies such as testicular, ovarian, lung and bladder cancer, Hodgkin and non-Hodgkin lymphoma [13,14]. Cisplatin is a very efficient chemotherapeutic agent, its toxicity, particularly nephrotoxicity and testicular damage limits its uses [11]. Cisplatin-induced testicular toxicity has been documented in several experimental studies [15,16]. Cisplatin-induced testicular toxicity underlying mechanism involves physiological and biochemical tissue disturbances resulting from oxidative stress and formation of ROS [17]. Progress in diagnostic modalities for assessing sperm function has refined the interpretation of events contributing to pathological spermatogenesis in idiopathic male infertility over the past few years [18]. Oxidative stress has been commonly investigated and found to play a detrimental role on sperm function [19]. ROS are oxygen-containing, chemically reactive molecules that are beneficial for optimal sperm functions such as the promotion of sperm capacitation, regulation of sperm maturation, and enhancement of cellular signaling pathways under normal physiological conditions [20]. Nonetheless, at higher levels, ROS have been shown to induce lipid peroxidation (LPO), sperm DNA damage, and abortive apoptosis [21]. To overcome these unwanted events, excessive ROS are naturally stabilized or deactivated by the body's antioxidant system [22].

D-ribose is an antioxidant and a pro drug form of l-cysteine known to aid the elevation of intracellular levels of glutathione (GSH) [23]. GSH is a coenzyme that mediates the protection against free radicals generated during the oxidative metabolism of acetaminophen by the hepatic cytochrome P-450 system [24]. GSH is the body’s most concentrated and ubiquitous antioxidant [25]. Oral supplementation with D-ribose-l-cysteine (DRLC) increases intracellular GSH in the liver, spleen, and other organs [26]. This study investigated ameliorative and fertility enhancing potential of nutritional dietary supplementation of D-Ribose –l-Cysteine in cisplatin induced oligoasthenoteratozoospermia and seminiferous epithelium degeneration in adult male Sprague-Dawley rats.

## 2 Materials and methods

### 2.1 Drugs and chemicals

Cisplatin, 5–5-dithio-bis (2-nitrobenzoic acid) (DTNB), thiobarbituric acid and reduced glutathione were purchased from Sigma-Aldrich Corp. (St. Louis, MO USA). All other chemicals and reagent used in this study were of analytical grade.

### 2.2 Preparation of D-Ribose-l-Cysteine solution

D-Ribose l-cysteine was obtained from Max International, Salt Lake City, Utah, USA. 2g of D-Ribose l-cysteine was dissolved in 400 ml physiological saline at 30 mg/kg body weight concentration of 1% solution.

### 2.3 Preparation of vitamin C

Vitamin C (manufactured by Emzor Pharmaceuticals, Nigeria) 500 mg of Vitamin C was dissolved in 100 ml of distilled water at concentration of 30 mg/kg body weight.

### 2.4 Experimental animals

Sixty (60) adult male Sprague-Dawley rats weighing between (150 and 200 g, 8weeks old) were bred in animal house of School of Basic Medical Sciences, College of Health Sciences, Federal University of Technology, Akure. The rats were housed in plastic cages in a well-ventilated room of natural photoperiod of about 12:12 h light darkness cycle at temperature: 27±10 ^O^C and 40-50% relative humidity) as prescribed by the United States National Institute for Health [27]. Fed with rat chow (Chikun Feeds Plc, Akure) and water *ad libitum*. The animals received humane care according to Care and Use of Laboratory Animals by National Academy of Science and National Institute of Health, and in compliance with ethical regulation of national and institutional guidelines for the protection of experimental animals’ right [28].

### 2.5 Experimental design

The rats were divided randomly into six (6) groups of ten (n = 10) rats each and treated as follows:

Group A, served as control and received 2.0 ml distilled water only throughout the treatment period.

Group B, received single dose of 8 mg/kg body weight (bwt) cisplatin only intraperitoneally.

Group C, received 30 mg/kg bwt DRLC only orally.

Group D, received single dose of 8 mg/kg cisplatin intraperitoneally follow by 30 mg/kg bwt DRLC orally.

Group E, received single dose of 8 mg/kg bwt cisplatin intraperitoneally and 50 mg/kg bwt of vitamin C orally.

Group F, received single dose of 8 mg/kg bwt of cisplatin intraperitoneally follow by 30 mg/kg bwt DRLC and 50 mg/kg bwt of Vitamin C orally. The experiment lasted for 56 days.

### 2.6 Collection of blood sample

Rats were sacrificed by cervical dislocation 24 h after the last administration under thiopental (100 mg/kg, i.p.). Before cervical dislocation, blood samples were collected between the hours of 8:00 a.m. and 10:00 a.m. into a plain sample tubes via orbital venous sinus with micro haematocrit tube. The blood was centrifuged at 2,500 g for 10 min to obtain serum samples within an hour after the blood collection. The sera obtained were later stored at -20^0^C till assayed for hormonal profile.

### 2.7 Determination of body, testicular weight and volume

The rats’ body weights was documented at procurement, acclimatization period, at the beginning of administrations, and once a week throughout the period of the experiment, by CAMRY electronic scale (EK5055, Indian). Testicular weight were estimated by weight of each rat’s testis and average value of the two testis taken as one observation in grams for body and testes weight. Testicular volumes were determined by water displacement method, volumes of the both testes were determined and average value obtained regarded as one observation, the values expressed as g/100 g body weight [29].

### 2.8 Preparation of testicular homogenate

The cleaned harvested testes were homogenized in ice-cold 0.25 M sucrose solution (1:5 w/v). The homogenates centrifuged at 10000*g* for 10 min at 4°C to obtain post-mitochondrial fractions and the resulting supernatant stored at -20°C to ensure maximum liberation of testicular fractions [30].

### 2.9 Determination of reproductive hormones

Assays for concentrations of testosterone, FSH, LH and prolactin were performed using the commercial enzyme immunoassay kits according to the manufacturer’s instructions (DRG Diagnostics GmbH, Marburg, Germany).

#### **2.9.1** **Determination of testicular 17** β **-HSD activity**

The testicular 17 β -HSD activity was evaluated as described Oyeyemi et al., [31]. The supernatant (1 mL) from homogenized testes was mixed with an equal volume of 440 μmol sodium pyrophosphate buffer (pH 10.2), 40 μL of testosterone (0.3 μmol) and 960 μL of 2.5% bovine serum albumin, to make a total of 3 mL incubation mixture. The 17 β-HSD activity was evaluated after adding 1.1 μmol nicotinamide adenine dinucleotide to the incubated mixture in spectrophotometer cuvette at 340 nm against a blank (without nicotinamide adenine dinucleotide). A unit of enzyme activity was equal to a change in absorbance of 0.001/min at 340 nm.

#### 2.9.2 Preparation of semen sample for analysis

A longitudinal surgical incision along the scrotal raphe and scrotal septum was made to expose the testes and its epididymis. The epididymis was freed from the adhering fat and connective tissues. The left epididymis was collected, weighed, and cut at the distal end using a clean surgical blade. Cauda epididymis (100 mg) was gently minced with glass rod without damaging the tissue into 5 ml of 0.9% NaCl [32].

#### 2.9.3 Determination of sperm motility, sperm count, and sperm morphology

Sperm motility was determined using the procedure by WHO [33]. A drop of prepared epididymal fluid was collected on a glass slide and covered with a coverslip (22 × 22mm) and examined under the light microscope (Olympia, Germany) immediately. The field was scanned systematically, and the motility of spermatozoa subjectively assessed and graded as progressive, non-progressive, and dead. At least 10 high power fields were observed at magnification of X400, and the relative percentage of spermatozoa in the different categories were estimated and recorded to the nearest 5% using the subjective method [33].

The epididymal sperm counts were obtained by the standard hemocytometer method. The epididymal fluid was thoroughly mixed with 10 mL of normal saline using a vortex, and approximately 10 μL of this diluted specimen was transferred to slides of the Bio-Rad counting chambers and counted with a Bio-Rad automated cell counter. Both sides of the counting chamber were used for each specimen and the average recorded to the nearest millions/milliliter [34].

Sperm morphology was accessed by staining dry smeared diluted epididymal fluid on a glass slide with eosin-nigrosine staining and observed under a light microscope (Olympia, Germany) at 400X magnification. The number of normal spermatozoa, spermatozoa with abnormal heads, spermatozoa with abnormal tail, and spermatozoa with abnormal midpiece was recorded in percentage.

#### 2.9.4 Determination of lipid peroxide levels

Blood hydroperoxide level was evaluated using an analytical system (Iram, Parma, Italy). The test is a colorimetric test that takes advantage of the ability of hydroperoxide to generate free radicals after reacting with transitional metals, when buffered chromogenic substance is added; a colored complex appears. This complex was measured spectrophotometrically. Lipid peroxidation level in the testis was measured by a method Ohkawa et al. [35] using thiobarbituric acid reactive substances (TBARS) with some modifications as previously described by Aboul-Soud et al. [36]. Testis was homogenized in ice cold 0.15 M KCl (10%) and the concentration of TBARS was expressed as nmol of MDA per mg protein using 1,1,3,3-tetramethoxypropane as standard. The absorbance was read at 532 nm.

#### 2.9.5 Determination of testicular glutathione content and activities of antioxidant biomarker enzymes

Testicular reduced glutathione (GSH) was determined using Ellman’s reagent 5-5-dithio-bis (2-nitrobenzoic acid) (DTNB) as a coloring reagent [37]. The absorbance was read at 412 nm by spectrophotometer. GSH concentration was calculated from a standard curve. The activity of the antioxidant enzyme testicular glutathione Peroxidase (GPX) was determined using glutathione reductase and NADPH. This method is based on the oxidation of NADPH at 25uC, which is indicated by the decrease in absorbance at 340 nm [38]. Results are expressed in U/mg protein. Testicular superoxide dismutase (SOD) was assayed by the method of Asada [39], which involves the inhibition of photochemical reduction of nitro blue tetrazolium (NBT) at pH 8.0. A single unit of enzyme is defined as the quantity of superoxide dismutase required to produce 50% inhibition of photochemical reduction of NBT. The absorbance was read at 580 nm against a blank using UV–Vis spectrophotometer. The activity was expressed as U/mg protein. Testicular Catalase (CAT) activity was estimated in testis homogenate by the method reported by Aebi [40]. The specific activity of catalase has been expressed as mmoles of H_2_O_2_ consumed/min/mg protein. The difference in absorbance at 240 nm per unit time is a measure of catalase activity.

#### 2.9.6 Histopathology procedure

The left testes were fixed in Bouin’s solution for 48 h, dehydrated in alcohol, and embedded in paraffin. Five micrometres thick sections were cut, and stained with haematoxylin and eosin (H&E). In a blinded manner, the slides were examined by a pathologist under light microscope (Olymia, Germany). Testicular injury was assessed by a semi-quantitative analysis for seminiferous epithelium damage, tubular necrosis, interstitial oedema, and haemorrhages using a scale from 0 to 3, where 0 means no abnormal findings, and 3 means severe abnormal findings [41]. Spermatogenesis was also assessed using a scale from 1 to 10, where 10 reflects normal spermatogenesis, and 1 reflects atrophy with no spermatogenesis, as previously described [42].

#### 2.9.7 Fertility test

Males’ rats were housed individually in a plastic cage with wood shavings as bedding. Three virgin females’ rats were placed inside the cage of one male for 2 h each day (7:00 to 9:00 h) and vaginal smears were evaluated for sperm splurge. The first 24-h period following the mating procedure was regarded as day 0 of pregnancy if sperm were detected in the smear. The mating procedure was repeated every working day until all three females became sperm-positive or, alternatively, for fifteen mating sessions extending over three weeks, mating and pregnancy index calculated as.

Mating index = [(No. of sperm-positive females)]/[(No. of mated females)]X100; Pregnancy index = [(No. of pregnant females)]**/**[(No. of sperm-positive females)] X100 [43].

#### 2.9.8 Statistical analysis

Data are expressed mean ± SEM (n = 10). One-way analysis of variance (ANOVA) followed by Tukey’s test was used for analyzing the statistical differences between different treatment groups using Graph pad prism 8 software. Level of significance was set at p < 0.05.

## 3 Results

### 3.1 Sperm parameters

**Fig. 1:**
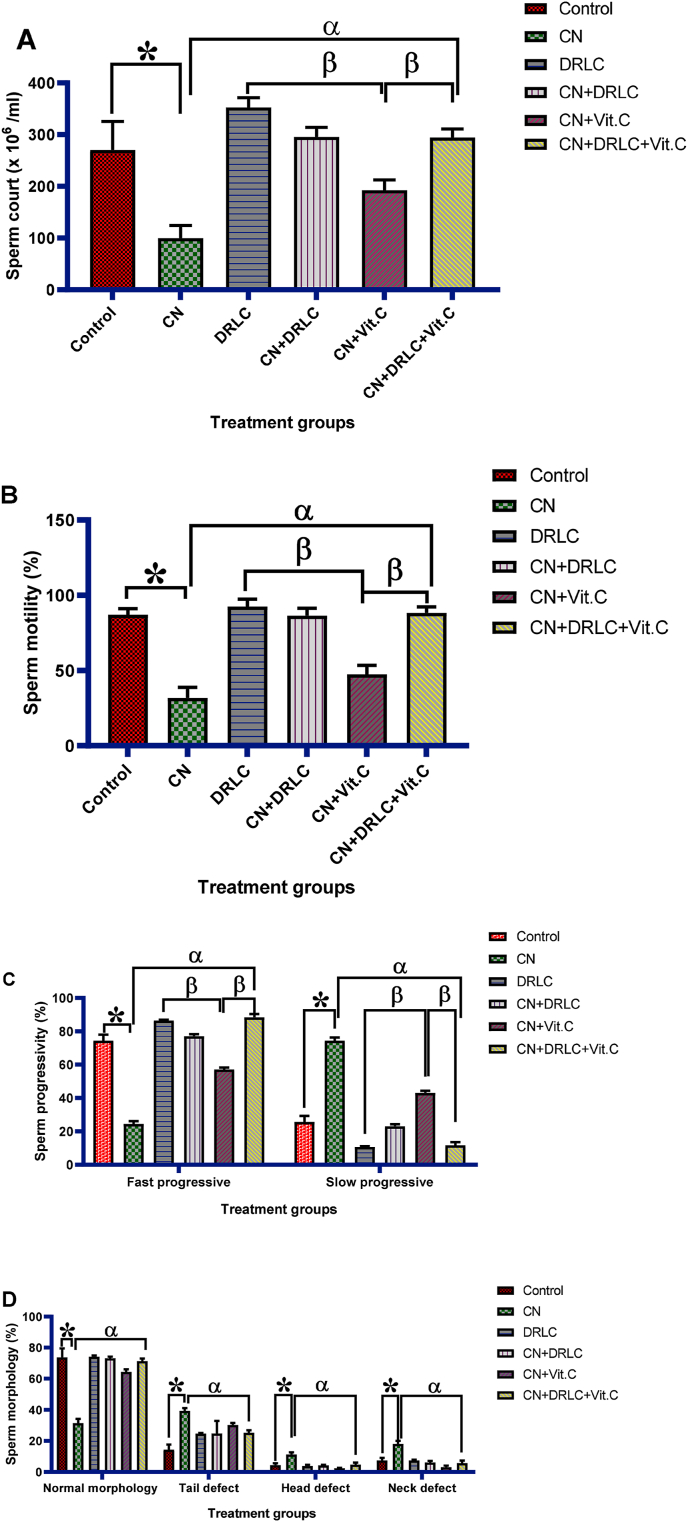
Effect of D-Ribose-l-cysteine on sperm count, motility, progressivity and morphology in cisplatin (CN) induced testicular toxicity. Data present as mean ± S.D., n = 10, *p<0.05 vs. control group, ^α^p<0.05 vs. CN group, ^β^p<0.05 vs. CN + Vit.C group. CN: Cisplatin, DRLC: D-Ribose-l-cysteine, Vit.C: Vitamin C.

D-Ribose-l-cysteine treatment insignificantly increase (p>0.05) the sperm quality as compared to control group. Administration of CN significantly decrease sperm count, sperm motility, fast progressive, percentage of sperm normal morphology and significant increase slow progressive and percentage of abnormal sperm morphology when compared to control group (p<0.05). Rats in the groups treated with CN + DRLC, CN + Vit.C and CN + DRLC + Vit.C exhibit a significant increase in sperm quality in comparison to that of CN treated rats (p<0.05). However, increase in sperm quality in groups treated with CN + DRLC and CN + DRLC + Vit.C showed significant different as compared to CN + Vit.C treated group [Fig. 1].

### 3.2 Hormones and testicular 17β-HSD activity

**Fig. 2:**
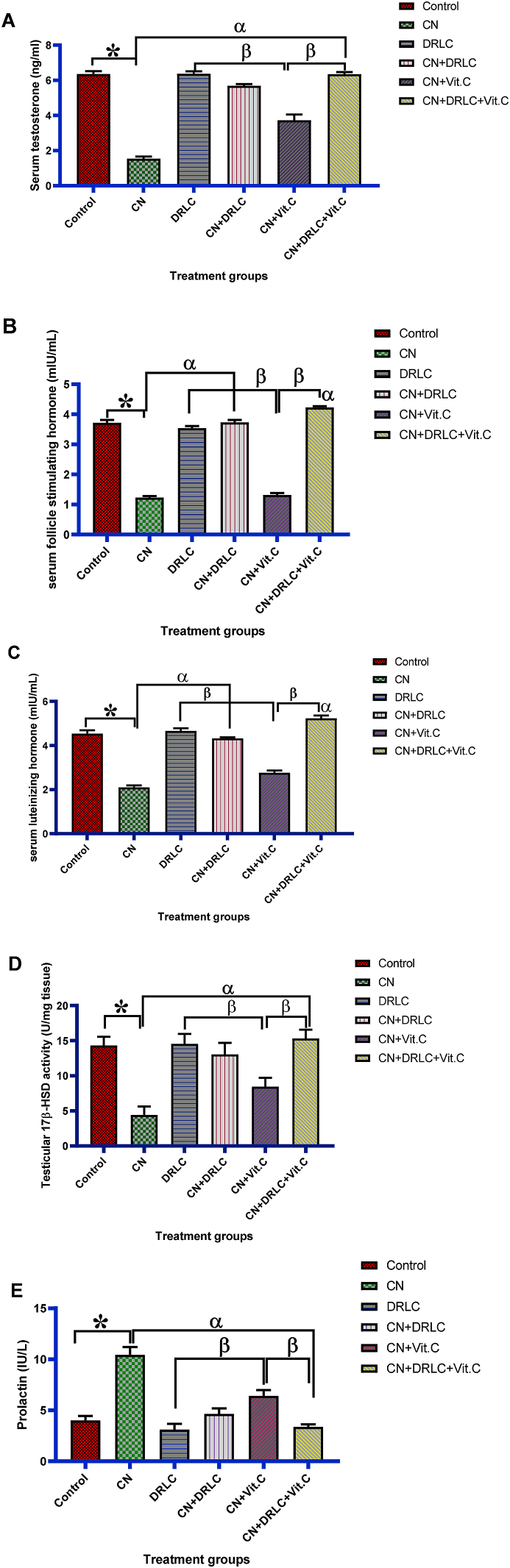
Effect of D-Ribose-l-cysteine on serum testosterone, follicle stimulating hormone, luteinizing hormone, testicular 17β-HSD activity and prolactin in cisplatin (CN) induced testicular toxicity. Data present as mean ± S.D., n = 10, *p<0.05 vs. control group, ^α^p<0.05 vs. CN group, ^β^p<0.05 vs. CN + Vit.C group. One way ANOVA. CN: Cisplatin, DRLC: D-Ribose-l-cysteine, Vit.C: Vitamin C.

Cisplatin exposure significantly reduced (p<0.05) the level of serum testosterone, FSH, LH and activity of testicular 17β-HSD and significantly elevate the prolactin level as compared to control group (p<0.05). Animals in the treated with DRLC alone showed no significant difference in hormones level and testicular 17β-HSD activity compared to control group. There was observed restoration of the hormonal level nearly in groups treated with CN + DRLC, CN + Vit.C and CN + DRLC + Vit.C nearly to that of control group and increase activity of testicular 17β-HSD significantly (p<0.05) in comparison to CN alone treated rats. Although, hormonal level and testicular 17β-HSD activity in CN + Vit.C treated group significantly decrease (p<0.05) when compared with of DRLC, CN + DRLC and CN + DRLC + Vit.C treated groups [Fig. 2].

### 3.3 Testicular lipid peroxidation products (MDA) and blood hydroperoxide level

**Fig. 3:**
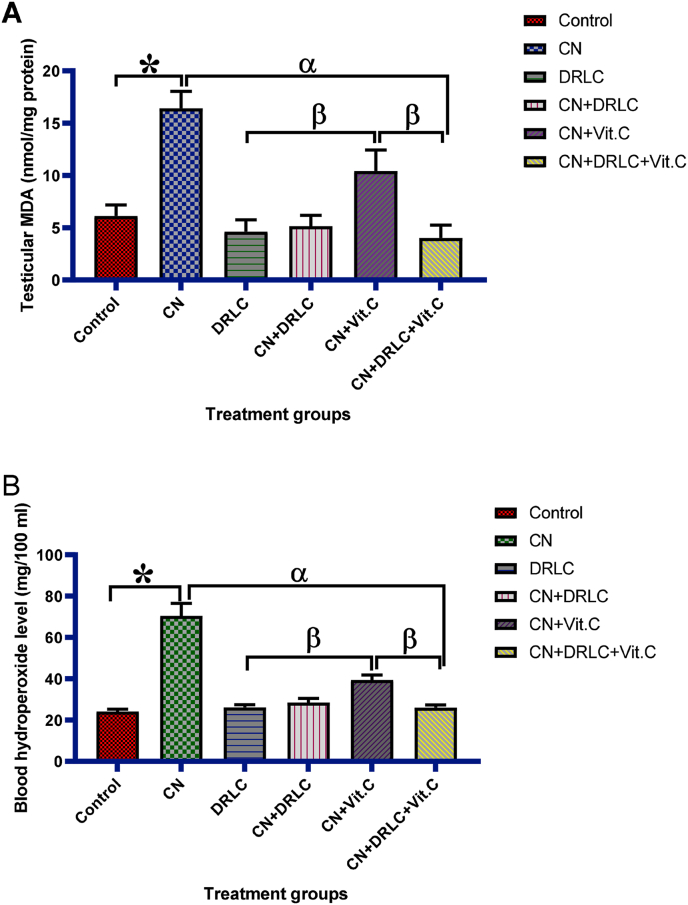
Effect of D-Ribose-l-cysteine on testicular lipid peroxidation products (MDA) and blood hydroperoxide level in cisplatin (CN) induced testicular toxicity. Data present as mean ± S.D., n = 10, *p<0.05 vs. control group, ^α^p<0.05 vs. CN group, ^β^p<0.05 vs. CN + Vit.C group. One way ANOVA. Cisplatin, DRLC: D-Ribose-l-cysteine, Vit.C: Vitamin C.

Testicular lipid peroxidation products (MDA) concentration and blood hydroperoxide level was increased significantly in CN exposed rats as compared to control group (p<0.05). However, administration of DRLC and co administration of CN and DRLC significant decrease the concentration of testicular MDA and blood hydroperoxide level compared with CN alone exposed rats (p<0.05). The concentration of testicular MDA and level of blood hydroperoxide in groups treated with CN + DRLC and CN + DRLC + Vit.C significantly reduce compared to CN + Vit.C treated group (p<0.05) [Fig. 3].

### 3.4 Testicular glutathione content and activities of antioxidant biomarker enzymes

**Fig. 4:**
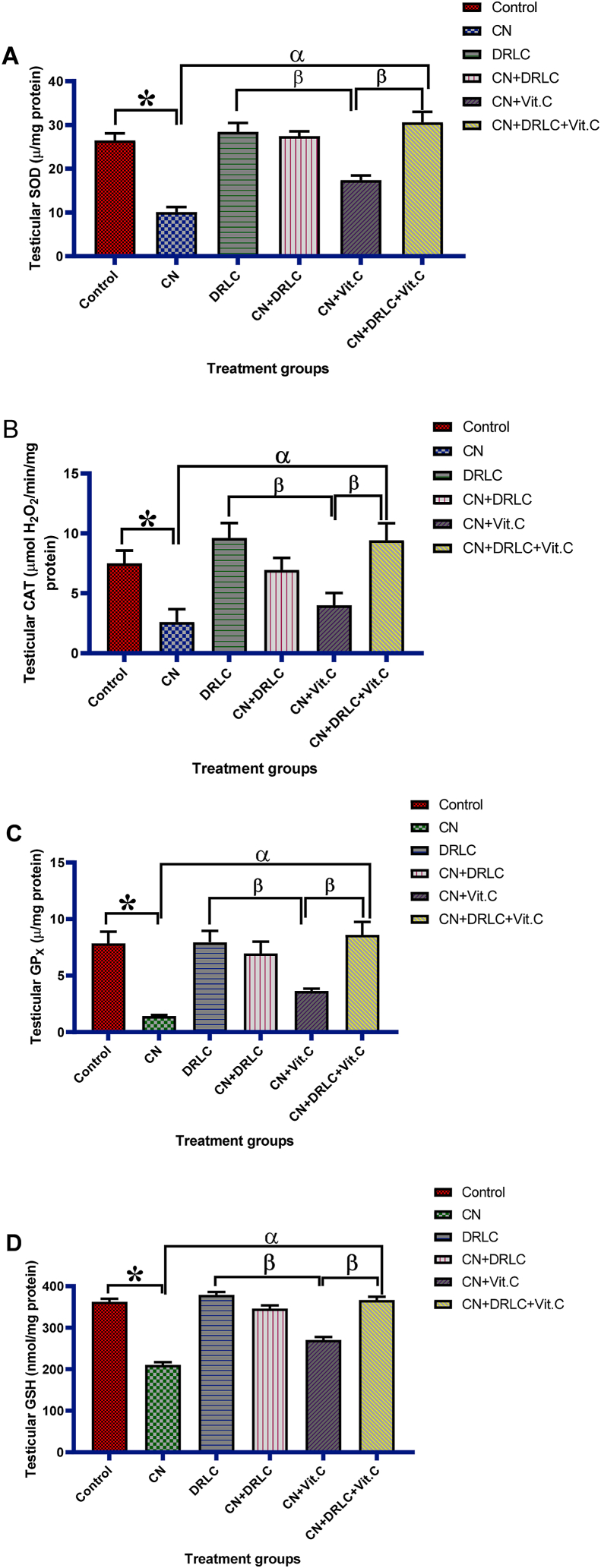
Effect of D-Ribose-l-cysteine on testicular glutathione content and activities of antioxidant biomarker enzymes in cisplatin (CN) induced testicular toxicity. Data present as mean ± S.D., n = 10, *p<0.05 vs. control group, ^α^p<0.05 vs. CN group, ^β^p<0.05 vs. CN + Vit.C group. One way ANOVA. Cisplatin, DRLC: D-Ribose-l-cysteine, Vit.C: Vitamin C.

Activities of SOD, CAT, GPx, and GSH concentration in the testis significantly reduce in CN expose animals as compared to that of control group (p< 0.05). There was significant increase in antioxidant activity in the testis of rats in group treated with DRLC alone compared to CN alone expose group (p<0.05). Testicular glutathione content and activities of antioxidant biomarker enzymes significant increase (p<0.05) in CN + DRLC, CN + Vit.C and CN + DRLC + Vit.C groups in comparison with CN exposed rats. Antioxidant enzymes (SOD, CAT, GSPx, and GSH) activities in testicular tissue was significant (p<0.05) higher in CN + DRLC and CN + DRLC + Vit.C groups than CN + Vit.C treated group [Fig. 4].

### 3.5 Body weight, testicular weight, testicular volume, diameter and cross-sectional area of seminiferous tubules

**Fig. 5:**
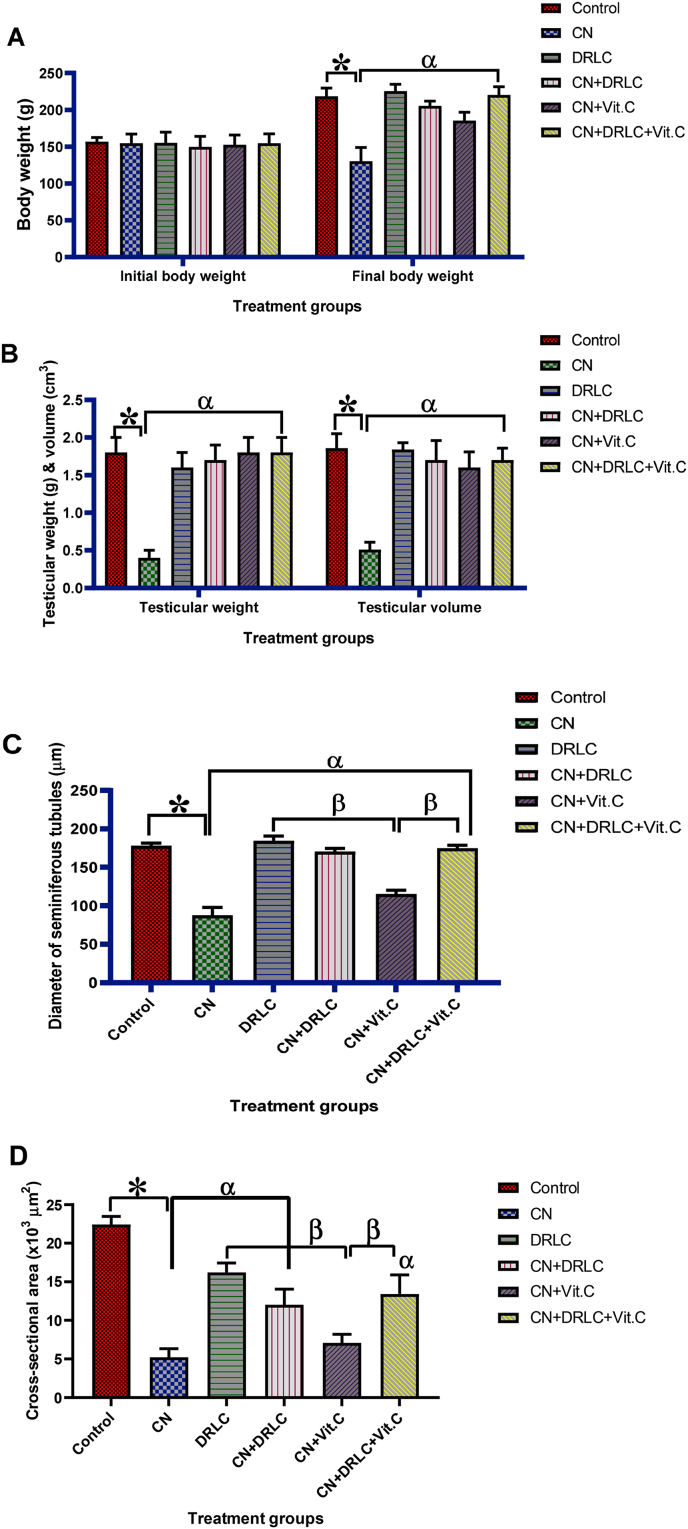
Effect of D-Ribose-l-cysteine on body weight, testicular weight, testicular volume, diameter and cross-sectional area of seminiferous tubules in cisplatin (CN) induced testicular toxicity. Data present as mean ± S.D., n = 10, *p<0.05 vs. control group, ^α^p<0.05 vs. CN group, ^β^p<0.05 vs. CN + Vit.C group. One way ANOVA. Cisplatin, DRLC: D-Ribose-l-cysteine, Vit.C: Vitamin C.

Observed significant loss (p<0.05) in body weight in CN exposed rats compared to control group. Similarly testicular weight and volume in CN exposed rats significantly reduced (p<0.05) as compared to control group. Post treatment with DRLC, Vit.C and DRLC + Vit.C showed significant increase in body weight, testicular weight and testicular volume compared with CN-alone injected rats (p<0.05). Also, treatment with DRLC alone revealed significant higher (p<0.05) value in body weight, weight of testis and testes volume than CN-alone treated group. The animals post treatment with DRLC and DRLC + Vit.C after CN-exposure showed insignificant higher value in body weight, testicular weight and volume than the animals post treated with Vit.C (p>0.05) [Fig. 5].

Administration of CN only significantly decrease (p<0.05) diameter and cross-sectional area of seminiferous tubules compared to control group. However, treatment with DRLC and DRLC + Vit.C significant increase the diameter and cross-sectional area of seminiferous tubules in comparison with CN only exposed rats. Post treatment with Vit.C showed no significant difference in cross-sectional area of seminiferous tubules as compared to CN alone treated rats. The groups treated with CN + DRLC and CN + DRLC + Vit.C had significant higher value in diameter and cross-sectional area of seminiferous tubules than CN + Vit.C treated group. Also, diameter and cross-sectional area of seminiferous tubules in DRLC alone treated animals had significant higher value than CN alone injected animals (p<0.05) [Fig. 5].

### 3.6 Testicular histology

**Fig. 6:**
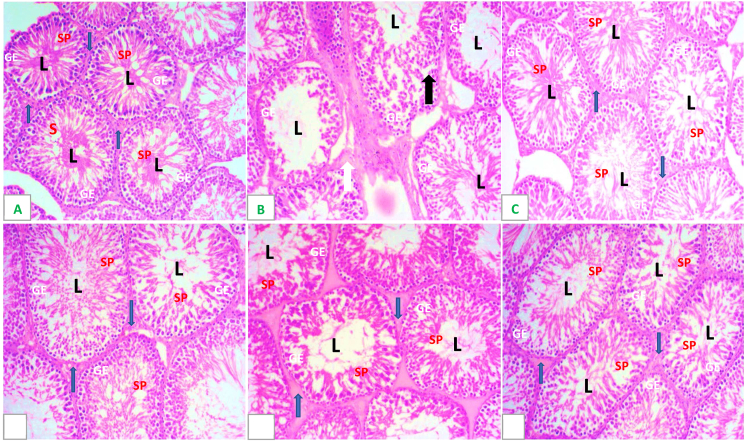
H&E (x200) photomicrographs of rat testes from: (A) control group showing normal testicular structure, germinal epithelium (GE) and spermatocyte (SP) and Interstitial/Leydig Cells (blue arrow); (B) cisplatin (CN) group showing necrosis of seminiferous tubular cells, desquamation of tubular epithelium (black arrow), vacuolization, absence of spermatogenesis, oedema of interstitium (white arrow);(C) Normal seminiferous epithelium with abundant sperm cells in the lumen (L) (D and E) respectively, showing marked improvement with minimal damage. (For interpretation of the references to color in this figure legend, the reader is referred to the Web version of this article.)

The testicular sections of control rats had normal histological profile characterized by hyperspermatozoa concentration in the lumen [Fig. 6A]. Testicular section of CN alone rats revealed degeneration and atrophied seminiferous tubules, interstitial oedema, degenerated and vacuolated germinal epithelium, absence of late stage germ cells, degenerated spermatogenic cells.[Fig. 6B]. Testicular section of DRLC alone reveal normal seminiferious epithelium with population of sperm cells in the lumen [Fig. 6C]. CN + DRLC, CN + Vit.C and CN + DRLC + Vit.C treated rats testis had a significant improvement in late stage germ cells and spermatogenic cells. The Leydig cells were evident with seminiferous tubular boundary evidently preserved. There was presence of sperm bundles radiating towards the seminiferous tubular lumen in most tubules [Fig. 6D–F].

### 3.7 Testicular injury and spermatogenesis score

**Fig. 7:**
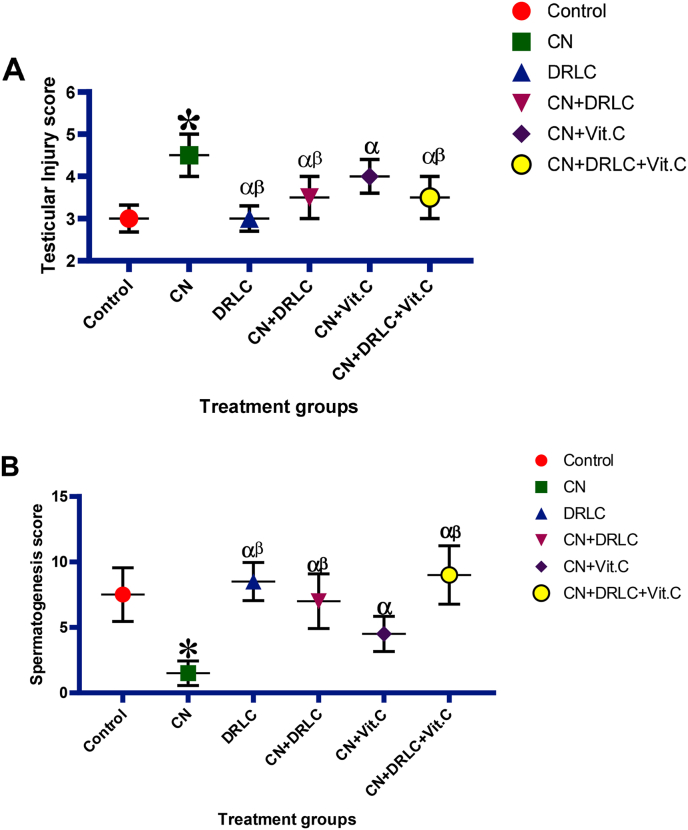
Effect of D-Ribose-l-cysteine on testicular injury score and spermatogenesis score in cisplatin (CN) induced testicular toxicity. Data present as mean ± S.D., n = 10, *p<0.05 vs. control group, ^α^p<0.05 vs. CN group, ^β^p<0.05 vs. CN + Vit.C group. One way ANOVA. Cisplatin, DRLC: D-Ribose-l-cysteine, Vit.C: Vitamin C.

Cisplatin exposure significantly increase (p<0.05) testicular injury scores and reduce spermatogenesis score compared to control. Post treatment with DRLC, Vit.C and DRLC + Vit.C resulted in significant reduction (p<0.05) in testicular injury score and boost spermatogenesis as compared with CN exposed group. However, the groups treated with CN + DRLC and CN + DRLC + Vit.C present significant lower (p<0.05) testicular injury score and higher spermatogenesis score than the group treated with CN + Vit.C. Administration of DRLC only showed no significant difference in testicular injury and spermatogenesis score as compared to control group but revealed significant decrease in testicular injury score (p>0.05) and significant increase in spermatogenesis when compared to CN alone injected group (p<0.05) [Fig. 7].

### 3.8 Fertility tests

Administration of CN alone present a significant adverse effect on fertility indices indicated by the proportion of females impregnated by male rats (mating index), and ratio of pregnant to sperm-positive females (pregnancy index) significantly different when compared to control and other treatment group. Post treatment with DRLC, Vit.C and DRLC + Vit.C significantly improved the mating and pregnancy index as compared to CN exposed rats. Administration of DRLC alone present positive fertility indices in both male and female rats compared to CN alone injected rats. Also, number of mating and pregnancy index is higher in DRLC, CN + DRLC and CN + DRLC + Vit.C treated rats than CN + Vit.C treated rats [Table 1].

**Table 1:** Effect of D-Ribose-l-cysteine on fertility index in cisplatin (CN) induced testicular toxicity.

Parameters	Treatment groups					
	Control	CN	DRLC	CN + DRLC	CN + Vit.C	CN + DRLC + Vit.C
No. of mated females	24	24	24	24	24	24
No. of mated males	8	8	8	8	8	8
No. of sperm-positive females	22	9	24	21	14	23
No. of pregnant females	17	3	20	18	12	22
% Mating index	92	38	100	88	58	95
% Pregnancy index	71	13	83	75	50	92

Data present as mean ± S.D., *p<0.05 vs. control group, ^α^p<0.05 vs. CN group, ^β^p<0.05 vs. CN + Vit.C group. Cisplatin, DRLC: D-Ribose-l-cysteine, Vit.C: Vitamin C.

### 3.9 Mechanism of action of D-Ribose-l-cysteine

**Fig. 8:**
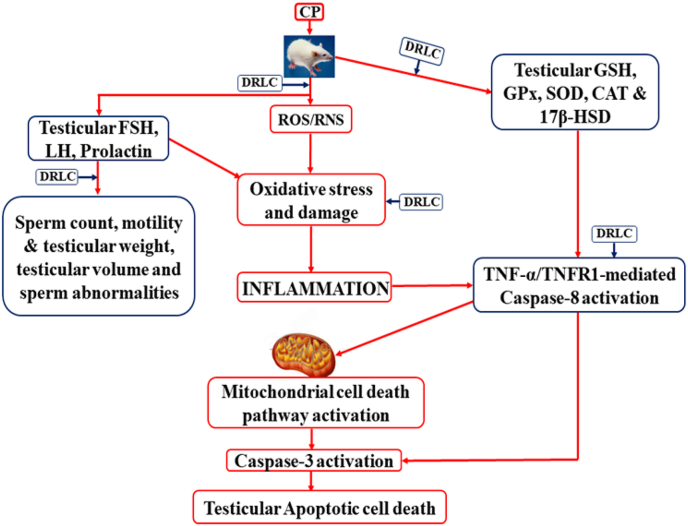
Schematic diagram of potential mechanism of action of D-Ribose-l-cysteine (DRLC) on cisplatin-induced toxicity.

Cisplatin induced overproduction of reactive oxygen species (ROS) and reactive nitrogen species (RNS), leading to oxidative stress in the testis. Oxidative lead to testicular inflammatory response (TNFα) increase and interact with TNFR1 to activate caspase-8, which activate caspase-3 either directly or through mitochondrial cell death pathway leading to apoptotic cell death. DRLC increase testicular antioxidant against oxidative stress, resulting in prevention of testicular inflammation and cell death [Fig. 8].

## 4 Discussion

Cisplatin (CN) is an effective antineoplastic drug that is employed as a front-line therapy against various cancers [44]. Cisplatin like other current chemotherapeutic agents is mostly non-selective in their efficacy, they kill rapidly dividing cells including cancer, normal and stem cells [1]. Oxidative stress could progressively damage the DNA and interfere with cell replication and transcription. Cisplatin also, suppresses the activity of antioxidant enzymes (GSH, SOD and CAT) and increases free radical levels (hydrogen peroxide and lipid peroxide) in the testes [45], all these processes are factors which are prerequisite to testicular degeneration. The therapeutic potential of D- Ribose-l-Cysteine (DRLC) in CN induced testicular degeneration was tested in this study, DRLC is the active ingredient in riboceine; one of the synthetic antioxidants that helps cell produce glutathione (GSH) on demand [46].

In this study, decreased testicular volume in CN alone exposed group could be associated with decrease in the number of Leydig cells when compared to other groups. High testicular volume in DRLC only treated group may be attributed to balanced homeostasis between reactive oxygen species and antioxidants defense system. While vitamin C (Vit.C) and DRLC + Vit.C post treated exhibited the protective role of Vit.C and riboceine. Furthermore, decrease in sperm motility, sperm concentration, sperm motile count, sperm percentage motility, total sperm concentration in CN injected rats is associated with impaired sperm production hence, lower testicular functions when compared with other groups corroborating the previous report of Franca et al. [47], that considerable germ cell damage by CN follow by a sharp degeneration in testicular parameters. Moreover, enhanced lipid peroxidation in CN treated rats, destroys the structure of spermatozoa accompanied by loss of motility and impairment of spermatogenesis [48].

Sperm morphology which includes the neck, tail and head was also assessed in this study. Cisplatin exposed rats had the least value in normal sperm morphology and highest value in tail, head and neck defect which may be attributed to increase concentration of ROS making the spermatozoa extremely liable to morphological defects due to the high content of poly unsaturated fatty acids in their plasma membrane similar to previous studies [49]. Administration of DLRC only recorded the highest value of normal sperm morphology and near normal morphological defects when compared to the control group emphasized the health benefits of DLRC in promoting spermatogenesis and steriodogenesis via the action of glutathione [50]. Abnormal sperm morphology in Vit.C and DRLC + Vit.C co-treated rats staged a near normal return to normal sperm morphology due to the antioxidative efficacy of DRLC and Vit.C in scavenging free radicals supporting previous observation on their protective effects [51,52].

Oxidative stress, a situation of imbalance between the free radicals (ROS) and antioxidant defense system, is an imperative cause in the pathogenesis of various ailments [53]. Estimation of end product of lipid peroxidation such as malondialdehyde (MDA) is an index of oxidative damage to cellular structures [54]. Significant increase in testicular MDA concentration in CN treated animals could be attributed to production of free radicals which buttresses previous studies that tissue level of MDA are proven indicators of oxidative stress resulting from lipid peroxidation [55] which in this study is caused by CN injection. Increase concentration of ROS subject the spermatozoa to impairment occasion by high level of polyunsaturated fatty acids in plasma membrane [49]. Moreover, it has been reported that enhanced lipid peroxidation in CN treated rats, destroys the structure of spermatozoa accompanied by loss of motility and impairment of spermatogenesis [56]. Post treatment with DLRC and Vit.C significantly decrease testicular activities of MDA, could be accrued to ameliorative and antioxidant capacity of DLRC and Vit.C. Glutathione (GSH) is an important antioxidant that protects the body from cellular damage caused by free radicals or excess ROS formation [57]. Decrease in activities of testicular CAT, SOD and GSH observed in CN exposed rats suggesting inability of testicular tissue to inactivate/dismutase O^-^2 and/or eliminate H_2_O_2_ thereby, leading to accumulation of highly reactive radicals within the testicular tissue [58]. The result agrees with previous studies [59] stating the depletion of CAT owing to an increase in H_2_O_2_ as a result of corresponding decrease in enzyme activity of free radicals creating an imbalance against the defense system which causes morphological disruption and loss of functional properties. The CN + DRLC treatment group produced a potential increase in depleted testicular antioxidant enzyme levels (GSH, SOD and CAT). D-Ribose-l-cysteine treatment significantly ameliorated the increased levels of oxidative stress markers, i.e. the mechanism behind DRLC protective antioxidant effect might be due to its antioxidant properties. Previous studies authenticated the prominence of oxidative stress in the pathogenesis of CN mediated gonadotoxicity [60,61].

In current observation, the hormonal assay showed a decrease level of Serum testosterone, LH, and FSH and this aids in making conclusions regarding reproductive pathologies. The production of testosterone in Leydig cells is stimulated by LH, which activates FSH to bind with Sertoli cells to stimulate spermatogenesis [62]. The outcome of the current investigation with reference to the testicular function marker enzymes in serum, revealed that CN exhibited a noteworthy suppression of testosterone, LH and FSH concentrations. The suppression of testosterone, LH and FSH by CN has been reported previously by Ref. [62], Garcia et al. [63], and Ilbey et al. [64], also reported that the inhibitory potential of CN on testosterone synthesis was due to ROS and also, revealed previously that antineoplastic agents can disrupt Leydig cells directly [65], leading to decrease serum testosterone. Steroidogenesis in the male rats is triggered by GnRH, which elicit the production and release of LH, which then binds to LH receptors on the membrane of Leydig cells to up regulate testosterone production [66]. This is in agreement with a previous study by Afsar et al. [67] on CN induced testicular toxicity. D-Ribose-l-cysteine treated groups showed an increase in the hormonal level corroborate the previous study on the effect of DRLC on infertility in wistar rats [68,69]. Observed a significant reduction in the testicular 17β-HSD activity CN exposed rats might be as a result of the decrease in LH, it is known that LH signals the initiation of steroidogenesis. Therefore, the reduction in testicular 17β-HSD activity in CN-injected rats might play a role in observed decrease in serum testosterone levels. Increased prolactin level in CN alone exposed rats associated with decreased testosterone level. This may be explained by the fact that CN increases prolactin mRNA expression [70] while hyperprolactinaemia alters testosterone production in rat testicular interstitial cells [71]. Administration of DRLC alone increase testicular 17β-HSD activity and decrease serum prolactin concentration. Post intervention of Vit.C and Vit.C + DRLC mitigate toxic effects of CN on gonadal axis.

Histological evaluation revealed distortion of the tubular architecture and disorganization of the spermatogenic cells in seminiferous tubules, hypo cellularity due to degeneration of germ cells, disruption of spermatogenesis and empty lumen in testes of rats submitted to CN injection, this is consistent with observations of Yucel et al. [72], that administration of CN leads to histopathological and structural changes in the testicular and tissues mostly evident in maturation loss in germinal cells and spermatogenesis arrest of primary spermatocytes. Also, these structural alterations in seminiferous epithelium could be attributed to oxidative stress expedited in deterioration of antioxidant defense system. However, post treatment with DRLC, Vit.C and DRLC + Vit.C showed total and notable restoration of the lumen seminiferous tubules with visible spermatozoa and abundant sperm cell, improved disrupted seminiferous epithelium could be link to antioxidant property of DRLC and Vit.C thereby, demonstrating positive effects on spermatogenesis and maintained structural integrity of testicular tissues.

In present study, our observation revealed significant decreased in rats testicular and body weight subject to CN injection, this is constant with the report of Garcia et al. [73], and Agu et al. [74], that exposure to CN occasion a reduced in reproductive organs and body weight since there is a serious health problem in patient undergoing CN chemotherapy are depressed appetite thereby, resulted in weight loss as a results of reduced feed efficiency. Acute exposure to chemotherapeutic drug such as CN can occasion reduction in reproductive organ weights, azoospermia, and degenerated spermatogenic cells [16]. Also, CN caused degeneration of seminiferous tubules and depletion of spermatogenic series in SD rats with a concomitant decrease in the diameter and cross sectional area of the seminiferous tubule with the presence of multinucleated cells in the lumen. This confirms the observation of several authors concerning the toxicity of CN [[74], [75], [76]]. This is could be link to arrested or altered spermatogenesis, especially with the decreased seminiferous epithelium diameter and cross sectional area. The decrease in seminiferous epithelium is attributed to the deleterious impacts of CN to increased ROS which occasion oxidative stress. Our result is also, in accordance with observation of Rekha et al. [77], who state that derangement in some testicular structures such as seminiferous tubules and Leydig cells could lead to about loss of 70% to 80% of testicular mass. Intervention of DRLC and Vit.C supplement increase the body weight and restore the distorted seminiferous epithelium, the increased is as a result of antioxidant capacity of DRLC and Vit.C. Testicular size and weight is one of the best primary assessments of spermatogenesis. Increase in testicular weight is mostly related to the number of spermatozoa present in the tissue [78].

Moreover, significant decrease in number of pregnant female rats after copulate with male rats in CN exposed rats confirmed oligospermia and asthenospermia in male rats since 95% of the male rats were unable to impregnate the female rats, this concur with our previous finding that CN alter male reproductive system since CN treated could not impregnate female rats after copulation [75]. However, intervention of DRLC and Vit.C + DRLC significantly ameliorate oligoasthenoteratozoospermia evidence in the proportion of females impregnated by male rats (mating index), and the ratio of pregnant to sperm-positive females (pregnancy index) and the significant improvement in fertility indices after post treatment with DRLC and Vit.C + DRLC could be related to strong antioxidant potential of DRLC and Vit.C + DRLC to fight against free radical generated by CN in testicular tissue thereby, induced oligoasthenoteratozoospermia and seminferious epithelium toxicity. Post treatment with Vit.C is less effective on fertility parameters compared with administration of DRLC alone and intervention of DRLC and Vit.C + DRLC after CN injection.

## 5 Conclusion

Cisplatin induced reproductive dysfunction at standard therapeutic dose levels can be protected by D-Ribose-l-Cysteine. Concurrent administration of cisplatin and D-Ribose-l-Cysteine. Could be encouraged to reduce the adverse reproductive effects of cisplatin when it is required for treatment of cancer in male.

## Ethical approval

All authors hereby, declare that all experiments have been examined and approved by the appropriate ethics committee and have therefore been performed in accordance with the ethical standards laid down in the 1964 Declaration of Helsinki.

## Funding

This research did not receive any specific grant from any funding agency in the public, commercial or not-for-profit sector.

## Declaration of competing interest

Authors have declared that no competing interests exist.
